# Left Atrial Coupling Index Predicts Heart Failure in Patients with End Stage Renal Disease

**DOI:** 10.3390/medicina60081195

**Published:** 2024-07-24

**Authors:** Fulya Avcı Demir, Gülsüm Bingöl, Mustafa Uçar, Özge Özden, Emre Özmen, Haşim Tüner, Muharrem Nasifov, Serkan Ünlü

**Affiliations:** 1 Department of Cardiology, Medical Park Hospital, 07160 Antalya, Turkey; 2 Department of Cardiology, Istinye University, 34010 Istanbul, Turkey; 3 Department of Cardiology, Istanbul Arel University, 34537 Istanbul, Turkey; bulut_gulsum@hotmail.com; 4 Department of Cardiology, Bahcelievler Memorial Hospital, 34180 Istanbul, Turkey; ozgeozdenctf@hotmail.com (Ö.Ö.); dremreozmen@yahoo.com (E.Ö.); hasimtuner@gmail.com (H.T.); drmeherrem@yahoo.com (M.N.); 5 Department of Cardiology, Celal Bayar University, 45140 Manisa, Turkey; mucar55555@gmail.com; 6 Department of Cardiology, Gazi University, 06570 Ankara, Turkey; unlu.serkan@gmail.com

**Keywords:** echocardiography, end stage renal disease, left atrial coupling index, preserved ejection fraction heart failure

## Abstract

*Background and Objectives:* We aimed to ascertain the predictive power of the left atrial coupling index (LACI) in patients with end stage renal disease (ESRD) for heart failure with preserved ejection fraction (HFpEF). *Materials and Methods:* This is a retrospective study including 100 subjects between 18 and 65 years of age with ESRD and not on dialysis treatment. Patients were divided into groups with and without HFpEF. The LACI was defined as the ratio of the left atrial volume index (LAVI) to the a′ wave in tissue Doppler imaging (TDI). Statistical analyses were performed, including univariate and multivariate regression analyses. *Results:* The mean age of the participants was 47 ± 13.3 years. Individuals with HFpEF exhibited a higher LACI. Univariate and multivariate regression analyses demonstrated that the predictive capacity of the LACI for HFpEF was considerably higher than that of the LAVI and other echocardiographic parameters. *Conclusions:* Higher LACI levels were consistently related to the presence of HFpEF in ESRD patients. The LACI can be easily obtained in daily practice using conventional Doppler echocardiographic measurements during left atrial functional assessments.

## 1. Introduction

Chronic kidney disease (CKD) is diagnosed when the glomerular filtration rate (GFR) is less than 60 mL/min/1.73 m^2^, regardless of the underlying cause, and when the albumin–creatinine ratio is ≥30 mg/g or when kidney damage is present for at least 3 months [1]. The World Health Organization has projected that CKD will become the fifth leading cause of chronic disease in twenty years [2]. CKD is staged in five groups. Stage IV of renal disease represents the advanced stage, in which the GFR is between 15 and 30 mL/min/1.73 m^2^, while Stage V represents the final stage, defined as end-stage renal disease (ESRD), in which the GFR is under 15 mL/min/1.73 m^2^. At this stage, preparations for renal replacement therapy should be initiated [1].

A number of studies conducted in recent years have demonstrated that the presence of CKD is a risk factor for the development of cardiovascular events (CVEs). The correlation between CKD and cardiovascular disease (CVD) was initially documented in 1836 by Richard Bright. It is estimated that 50% of all patients with CKD stages 4 to 5 have CVEs [3]. Within the context of CVEs in these patients, heart failure (HF) represents the most prevalent cause of death, followed by stroke and myocardial infarction. The most recent data on the current guidelines indicate that approximately half of patients diagnosed with HF have a preserved left ventricular ejection fraction (LVEF). Heart failure with preserved ejection fraction (HFpEF) is diagnosed when a patient presenting with HF signs and symptoms has a LVEF equal or greater than 50% and evidence of functional or structural heart disease. Additionally, the presence of elevated natriuretic peptide levels and/or functional or structural cardiac abnormalities is required [4]. Consequently, it is of critical importance to conduct comprehensive assessments and to provide close monitoring of cardiac functions in patients with CKD, particularly in those who have ESRD, in the most vulnerable stage. It is known that transthoracic echocardiography (TTE) plays an essential role in cardiology to assess cardiac functions in detail.

In HFpEF, it is postulated that left atrium (LA) dysfunction is the primary cause of LV diastolic dysfunction and HF symptoms [5]. Although numerous hemodynamic parameters, including pulmonary vein flow and mitral inflow, LA volume, and LA volume index (LAVI), are currently employed for the assessment of LA functions, the left atrial coupling index (LACI) represents a novel and intriguing parameter. The LACI was obtained from the division of the LAVI to the medial mitral annulus a′ velocity (LAVI/a′) by tissue Doppler imaging (TDI) [6]. The prognostic efficacy has been evaluated in cases of HF with low ejection fraction, atrial fibrillation, and mitral regurgitation. Higher LACI values were associated with a poorer prognosis [7,8].

According to our knowledge, to date, no study has examined the potential role of the LACI as a predictor of HFpEF in ESRD patients. The aim of our study was to investigate the predictive value of the LACI in identifying HFpEF in non-dialyzed ESRD patients.

## 2. Materials and Methods

### 2.1. Study Design and Population

This retrospective study included 100 subjects with ESRD, aged 18 to 65 years, who were referred to the outpatient cardiology clinic for preoperative evaluation for renal transplantation between February 2023 and April 2023. None of the patients were receiving dialysis treatment. Clinical and demographic data including etiology of CKD, New York Heart Association (NYHA) functional class, laboratory values, medications, physical examination, and TTE findings of the patients were obtained from the national health system data and hospital database. Patients younger than 18 years old, a CVE within the previous 3 months, with acute infection, with a history of transplantation, active malignancy, cirrhosis, morbid obesity, asthma, chronic obstructive lung disease, congenital heart diseases, pulmonary hypertension, moderate or severe valvular disease, arrhythmias, ongoing pregnancy, or left or right ventricular dysfunction were excluded.

### 2.2. Echocardiographic Assessment

All participants were subjected to a comprehensive TTE examination utilizing a Vivid S5 (GE Vingmed Ultrasound AS, Horten, Norway) by an experienced cardiologist who performed the echocardiography procedure according to the latest guidelines [9]. The assessments were based on the analysis of recorded images. Echocardiographic examinations were performed in the left decubitus position at rest, including standard two-dimensional imaging, M-mode, and TDI and pulsed wave evaluation at the septal and lateral mitral annulus. The Simpson method was used for calculating the LVEF. TDI and pulsed wave Doppler measurements, including the transmitral early (E) and late diastolic peak flow velocities (A) and their ratio (E/A), were obtained from the apical four-chamber view. The average of the peak early diastolic relaxation velocities (e′) of the lateral and septal mitral annulus was calculated. The LAVI was figured out by dividing the LA volume by the body surface area. The LAVI was defined as elevated if it was greater than 34 mL/m^2^. The LACI was obtained by dividing the LAVI to a′ velocity at the medial mitral annulus.

### 2.3. Statistical Analysis

While categorical data are presented as frequencies or percentages, continuous variables are presented as mean ± standard deviation. The Kolmogorov–Smirnov test was employed to assess the distribution of continuous variables in order to ascertain their normality. The Student *t*-test and Mann–Whitney U test were employed to contrast nonparametric and parametric continuous variables, respectively. The patients were randomized into two groups, one comprising those with HFpEF and the other comprising those without HFpEF. The Chi-square (χ^2^) test was used to compare the categorical variables. Univariate and multivariate linear regression analyses utilizing contributors with a significant correlation and conventional clinical variables (age) were entered in a backward stepwise logistic regression model. In order to evaluate the predictive accuracy of HFpEF diagnosis, receiver operating characteristic (ROC) curves, the Youden index, and the area under the curve (AUC) were calculated (Medcalc Software, Version 15.8, Mariakerke, Belgium). A two-tailed *p*-value less than 0.05 was considered to be statistically significant. SPSS v23.0 (IBM Corp, Armonk, NY, USA) programme was used to analyze the data.

### 2.4. Ethical Considerations

This study was approved by Ethics Committee of Memorial Hospital, Istanbul, Turkey, approval Date: 5 January 2023, approval Code: 85. The study was conducted under the guidelines of the Declaration of Helsinki and the principles of Good Clinical Practice and respect for the rights and dignity of all parties. Verbal and written informed consent forms were obtained from participants after a detailed explanation of the aim of the study.

## 3. Results

One hundred patients with ESRD were included in the study. According to the presence of HFpEF diagnosis, patients were divided into two groups. There were 47 patients (47%) with ESRD who were also diagnosed with HFpEF. Table 1 provides an overview of the co-morbidities, baseline characteristics, and laboratory findings of the study group. Coronary artery disease and age were found to be statistically significant in favor of the group with HFpEF. (<0.05). Notably, several laboratory parameters exhibited statistically significant differences between the two groups of patients. These included lactate dehydrogenase (203.9 ± 62.2 vs. 233.4 ± 70.4; *p* = 0.028), Ca (9.1 ± 0.6 vs. 8.7 ± 0.8; *p* = 0.016), and Mg (2.1 ± 0.3 vs. 1.9 ± 0.4; *p* = 0.048). No statistically significant differences were observed in the remaining clinical, demographic, and laboratory parameters (Table 1).

Considering all TTE parameters, LV diameters and volumes were observed to be higher in patients with HFpEF. LV ejection fraction was statistically significantly higher in patients without HFpEF compared to those with HFpEF. Additionally, LA was larger (3.6 ± 0.5 vs. 4.2 ± 0.6; *p* < 0.001), and the LACI and LAVI values were greater (4.1 ± 1.5 vs. 9.2 ± 4.8; *p* < 0.05 and 34.3 ± 11.6 vs. 51.3 ± 13.3; *p* < 0.05, respectively) in patients with HFpEF. RV functions, assessed with TAPSE and FAC, were similar between the two groups (Table 2).

To identify the predictors of HFpEF, univariate and multivariate logistic regression analyses were conducted. The analysis results revealed that a high LACI level is independently correlated with the presence of HFpEF in ESRD patients. (OR, 2.824; 95% CI, 1.776–4.491; *p* < 0.001) (Table 3).

**Figure 1:**
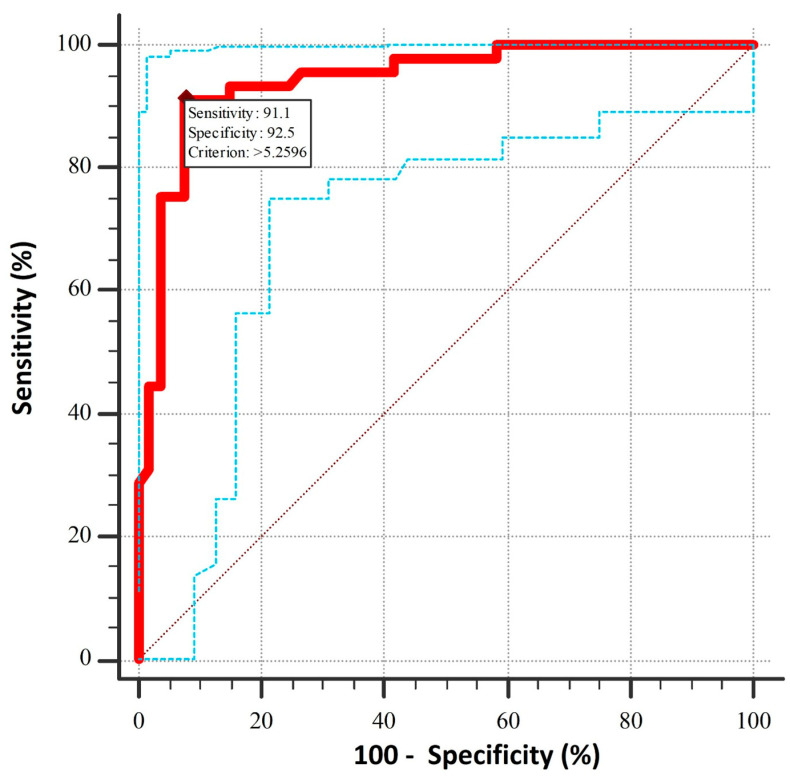
Receiver operating curve analysis for LACI for the prediction of HFpEF (▬: receiver operating curve, **…**: upper- and lower 95% confidence interval boundaries of the receiver operating curve).

The ROC analysis of the LACI (AUC = 0.94: 95% CI: 0.87–0.98) demonstrated a high degree of accuracy in the prediction of HF diagnosis, with a specificity of 92.5% and a sensitivity of 91.1% for a cut-off value of 5.23 (Figure 1).

## 4. Discussion

The results of our study indicate that a higher LACI value, which is a recently developed echocardiographic parameter that can be readily employed to assess LA dysfunction, is significantly correlated with the presence of HFpEF in non-dialyzed ESRD patients.

CKD is a nephrological syndrome characterized by irreversible, progressive, and chronic loss of nephrons due to various diseases. Cardiovascular events (CVEs) are a major cause of mortality among CKD patients [10]. CV mortality accounts for approximately 40% to 50% of all deaths in patients with advanced CKD (stage 4) and end-stage renal disease (stage 5), compared with 26% in controls with normal kidney function. In addition to the elevated risk of fatal complications associated with atherosclerosis, such as myocardial infarction and stroke, cardiovascular deaths also occur due to heart failure (HF) and fatal arrhythmias, particularly in advanced stages [3].

The frequent occurrence of cardiovascular diseases in patients with CKD can be attributed to the coexistence of various cardiovascular risk factors, both traditional (hypertension, diabetes, dyslipidaemia, advanced age) and non-traditional (volume overload, abnormalities in mineral metabolism, anaemia, malnutrition, volume overload, oxidative stress, inflammation, and proteinuria) [11]. A meta-analysis, which included over 100,000 individuals from the general population, demonstrated that both a reduction in eGFR and the presence of albuminuria were independently and strictly associated with an increased risk of death (including CV death), irrespective of numerous potential confounding factors, such as gender, age, and traditional CV risk factors [12]. In over 70 studies of individuals with CKD not on dialysis, the correction of classical and even less classical cardiovascular risk factors, such as hypertension, diabetes, and dyslipidemia, did not neutralize the impact of CKD on cardiovascular risk [3]. Given the correlation between cardiovascular mortality and morbidity and renal function, ESRD represents a period of high vulnerability for patients with cardiovascular pathologies, particularly coronary artery disease and heart failure. The prevalence of HF represents a significant cause of morbidity and mortality in patients with ESRD [13]. Approximately half of the patients diagnosed with HF are in the HFpEF group. In recent years, there have been notable advancements in the diagnosis and treatment of patients with HFpEF, which is of paramount clinical importance. The pathophysiology of HFpEF includes a range of underlying diseases, including renal disease, diabetes, hypertension, atrial fibrillation, and coronary artery disease [14]. In a previous study, according to their phenotype, patients with HFpEF were divided into three groups. The first group consisted of younger, predominantly female patients with low levels of natriuretic peptides and less cardiac remodeling. The second group included obese, diabetic, and predominantly female patients with LV dysfunction. The third group comprised older patients with CKD. The adverse cardiovascular end points were found to be more common in the group of older patients with CKD [15].

The results of our study demonstrated a statistically significant higher LV ejection fraction in the patient group without HFpEF compared to the patient group with HFpEF. However, we believe that this statistical difference is not clinically significant. In both groups of patients included in the study, the ejection fraction was within the normal range. Even though the classification of HF primarily focuses on the LV, it is not possible to assess left ventricular function and diagnose HFpEF solely on the basis of a single echocardiographic measure. It is important to note that the diagnosis of heart failure with preserved ejection fraction is based on a comprehensive clinical evaluation, taking into account the presence of relevant clinical symptoms and signs.

HFpEF is intimately associated with a prognostic disadvantage and consists of a mixture of pathophysiological changes including diastolic dysfunction, LV and LA remodeling, pulmonary vascular hemodynamics, and some noncardiac factors. In patients with HFpEF, the function of the LA also plays a crucial role in the identification of novelly validated, structural, and functional parameters into a diagnostic score; this may better define this heterogeneous disorder [14].

LV diastolic dysfunction causes remodeling and dysfunction due to changes in LA volume and compliance. LA volume and compliance serve as important indicators of the severity and duration of diastolic dysfunction, irrespective of loading conditions. Given that LA does not expand equally in all directions, the body surface area-indexed measurement of the maximal LAVI, which has been demonstrated in numerous studies to be closely related to cardiac endpoints and patient prognosis, is considered a superior method to anteroposterior diameter measured via the parasternal long axis window [16]. According to the most recent guidelines, the LAVI should obtained by indexing the LA volume, which is measured from apical two and four-chamber views to body surface area. The maximum LAVI considered normal is 34 mL/m^2^ using 2D echocardiography tests. An increased LA volume due to diastolic dysfunction is widespread in patients with HFpEF and is related to a worse prognosis. LA dysfunction is the primary cause of LV dysfunction and HF symptoms in HFpEF patients [5,14]. To assess LA function, various hemodynamic parameters such as pulmonary vein flow, mitral inflow, peak velocity of tricuspid regurgitation, and the LAVI are used [4,5]. The reliability of the LAVI and the E/e′ in detecting diastolic dysfunction has been corroborated by a number of studies [17,18,19]. E/e′ has been demonstrated to correlate well with both pulmonary capillary wedge pressure and LV filling pressure [17,18], whereas the LAVI indicates decreased LV compliance due to chronic diastolic dysfunction [19]. Despite the guidelines recommending E/e′ and the LAVI as the most effective parameters for detecting diastolic dysfunction, our study demonstrated that the LACI outperforms these parameters, emerging as the sole significant parameter in the multivariate analysis [14].

LA strain analysis is a valuable tool for understanding the pathogenesis of HFpEF, as well as for making a diagnosis and determining prognosis [20]. The LA strain, particularly the peak atrial longitudinal strain (PALS), is a measure that can detect LA dysfunction early when other echocardiographic parameters used to assess diastolic dysfunction are completely normal [21]. The current recommendations indicate that the evaluation of LA functions should be conducted by utilizing the LAVI in conjunction with the LA contractile or reservoir strain in patients with HFpEF [22,23,24].

Nevertheless, for LA strain analysis to be accurate and reliable, the patient’s image quality must be of a high standard, there must be an appropriate software program for the analysis, and a cardiologist or an echocardiographer must be trained and experienced in strain assessment.

The LACI represents a novel parameter that combines the volumetric and mechanical properties of the LA. The ratio of the LAVI to tissue Doppler a′ (a′-TDI) is called the LACI. It can be readily measured and reproduced in routine clinical practice. The LACI has certain advantages over E/E′ and the LAVI. A brief summary of these advantages reveals that E/E′ reflects changes in filling pressure in the acute phase, whereas the LACI is more reliable in the evaluation of chronic diastolic dysfunction [25]. Furthermore, the LACI offers the opportunity to evaluate LV filling pressures and compliance simultaneously, and is less affected by age [26].

The results of studies conducted with the LACI have yielded promising outcomes. In a study of 395 patients with dyspnea and NYHA class 2–4 functional capacity, Park et al. determined that the optimal value for the LACI ratio was 4.0 in order to define diastolic dysfunction. A 31.9-month follow-up revealed that the incidence of readmission due to HF and/or cardiac death was remarkably higher in the LACI ≥ 4.0 group than the LACI < 4.0 group (*p* < 0.001). According to these findings, it was concluded that a LACI value ≥ 4.0 is an independent determinant of clinical outcomes [26]. In a large cohort study, Essayagh et al. demonstrated that a LACI value exceeding 5 was a robust and consistent predictor of mortality, independent of the severity of mitral regurgitation and LA size in patients with floppy mitral valves [7].

The objective of our study was to ascertain whether the LACI, which is more accessible and easier to calculate, can be employed in clinical practice as an additional parameter in diagnosing HFpEF in the ESRD patient population, in conjunction with existing diagnostic tools. It is first and foremost important to bear the diagnosis of HFpEF in mind. The identification of the LACI as a predictor of HFpEF in ESRD patients may prove beneficial in establishing the diagnosis and organizing treatment rapidly, identifying patients who could potentially benefit from treatment and close follow-up, and ultimately improving the prognosis in these individuals. The diagnosis of HF using traditional biomarkers such as natriuretic peptides and troponins is challenging due to the fact that the threshold is not valid in the presence of CKD [4].

## 5. Limitations

The findings of our study are of value, but it is important to recognize the potential limitations of the study. It is possible that diagnostic tests and invasive procedures were performed with greater frequency in those who subsequently developed heart failure. This may have resulted in an increased frequency of coronary artery disease diagnosis within this group, potentially introducing a degree of diagnostic bias. In addition to that, our retrospective study results could be enhanced by the further investigation of dynamic changes in the LACI in ESRD patients. This could be achieved through larger-scale, multicenter prospective studies that focus on investigating changes before and after transplantation. Moreover, studies with a period of extended observation and monitoring could be designed to analyze the impact of the LACI on mortality and long-term morbidity.

## 6. Conclusions

We found that regardless of baseline characteristics, the LACI independently and strongly predicts the presence of HFpEF in patients with ESRD. Further prospective studies are required to examine the potential changes in the LACI in this patient group and substantiate these findings.

## Figures and Tables

**Table 1:** The clinical and laboratory characteristics and baseline demographics of the patients.

	Patients without HFpEF (n = 53)	Patients with HFpEF (n = 47)	*p*-Value
Age (years)	43.3 ± 14.3	49.9 ± 12.3	0.016
Sex (f, %)	34 (64.2)	28 (59.6)	0.683
BSA (m^2^)	1.7 ± 0.2	1.7 ± 0.2	0.708
Duration of renal disease (years)	7 ± 5.3	7.2 ± 7.2	0.840
Hypertension (n, %)	37 (69.8)	41 (87.2)	0.052
Diabetes Mellitus (n, %)	13 (24.5)	17 (36.2)	0.147
Smoking (n, %)	18 (34)	12 (25.5)	0.390
Family History (n, %)	7 (13.2)	3 (6.4)	0.328
CAD (n, %)	4 (7.5)	14 (29.8)	0.004
Hemoglobin (gr/dL)	10.9 ± 1.8	10.8 ± 2	0.813
Parathyroid hormone (pg/mL)	167 ± 142.8	175.1 ± 148.4	0.879
Creatinine (mg/dL)	7.7 ± 3.4	6.8 ± 2.7	0.141
Lactate dehydrogenase (U/L)	203.9 ± 62.2	233.4 ± 70.4	0.028
Na (mEq/L)	135.4 ± 19.2	135.2 ± 20.3	0.947
Mg (mg/dL)	2.1 ± 0.3	1.9 ± 0.4	0.048
K (mmol/L)	4.8 ± 0.9	4.8 ± 0.6	0.984
P (mg/dL)	5 ± 1.5	4.9 ± 1.8	0.592
ALT(IU/L)	16.5 ± 13.5	14.3 ± 8.3	0.339
Total Protein (g/dL)	7.2 ± 1	6.9 ± 0.7	0.107
Ferritin (ng/mL)	453.9 ± 374.8	518.5 ± 474	0.449
TSH (mIU/L)	1.8 ± 1.1	2.2 ± 1.5	0.097
Uric Acid (mg/dL)	6 ± 2.1	6.1 ± 1.8	0.693
Glucose (mg/dL)	115.5 ± 59.4	121.6 ± 51.1	0.584
Albumin (g/dL)	4.3 ± 0.5	4.2 ± 0.5	0.301
C-Reactive Protein (mg/dL)	14.8 ± 22.9	17.1 ± 26.1	0.642
Ca (mg/dL)	9.1 ± 0.6	8.7 ± 0.8	0.016

BSA: Body surface area, ALT: Alanine Aminotransferase, CAD: coronary artery disease, Mg: magnesium, K: potassium, Na: sodium, P: phosphor, Ca: calcium, TSH: Thyroid Stimulating Hormone.

**Table 2:** Echocardiographic measurements of the groups.

Parameters	Patients without HFpEF (n = 53)	Patients withHFpEF (n = 47)	*p*-Value
Dimensions, areas, and volumes of left ventricle			
LV end-diastolic diameter (cm)	4.5 ± 0.6	5.1 ± 0.6	<0.001
LV end-systolic diameter (cm)	2.9 ± 0.4	3.1 ± 0.5	0.004
LV end-diastolic volume (mL)	97 ± 34.5	118.9 ± 42	0.005
LV end-systolic volume (mL)	33.3 ± 11.8	47.2 ± 27.2	0.001
LV ejection fraction (%)	66.5 ± 4.5	62.6 ± 9.8	0.011
Dimensions. areas, volumes of left atrium			
LA diameter (cm)	3.6 ± 0.5	4.2 ± 0.6	<0.001
LA volume (cm^3^)	58.7 ± 18.8	90 ± 25.9	<0.001
LA volume index (mL/m^2^)	34.3 ± 11.6	51.3 ± 13.3	<0.001
Left Atrioventricular Coupling Index (mL/m^2^)	4.1 ± 1.5	9.2 ± 4.8	<0.001
Doppler measurements of left ventricle			
E (cm/s)	0.8 ± 0.2	0.9 ± 0.3	0.008
A (cm/s)	1 ± 0.6	0.9 ± 0.2	0.357
IVRT (ms)	91.7 ± 25.9	101 ± 27.5	0.086
Deceleration time (ms)	147.3 ± 38.1	164.1 ± 58.6	0.090
Tissue Doppler measurements of left ventricle			
Lateral e′ (cm/s)	10.4 ± 4.6	7.7 ± 3.8	0.002
Lateral a′ (cm/s)	10.2 ± 2.8	7.4 ± 2.7	<0.001
Lateral s′ (cm/s)	9.1 ± 2.5	7.3 ± 2.4	<0.001
Septal e′ (cm/s)	7.5 ± 3	6.6 ± 2.3	0.102
Septal a′ (cm/s)	8.7 ± 2	6.3 ± 1.9	<0.001
Septal s′ (cm/s)	6.7 ± 1.2	5.9 ± 1.2	0.001
E/e′	9.8 ± 4.6	13.9 ± 4.5	<0.001
Functional assesment of right ventricle			
RV FAC (%)	58.4 ± 5.3	58.5 ± 5.9	0.964
TAPSE (cm)	2.3 ± 0.4	2.3 ± 0.4	0.561

LV; left ventricle, FAC; fractional area change, RV: right ventricle, LA; left atrium, TAPSE; tricuspid annular plane systolic excursion.

**Table 3:** Univariate and multivariate linear regression analysis of predictors for heart failure.

Variables	Univariate				Multivariate			
			95% Confidence Interval				95% Confidence Interval	
	B	*p*-Value	Lower	Upper	β	*p*-Value	Lower	Upper
Sex (Female)	1.014	0.671	0.950	1.083	-	-	-	-
Age	4.498	0.069	0.888	22.782	-	-	-	-
Duration of CRD	1.044	0.656	0.863	1.264	-	-	-	-
Smoking	5.838	0.096	0.730	46.670	-	-	-	-
Hypertension	0.222	0.152	0.028	1.743	-	-	-	-
Diabetes	1.411	0.714	0.225	8.853	-	-	-	-
CAD	0.212	0.233	0.017	2.709	-	-	-	-
Family History	0.686	0.697	0.103	4.573	-	-	-	-
LV EF	0.850	0.086	0.706	1.023	-	-	-	-
LAVI	1.019	0.723	0.919	1.130	-	-	-	-
LACI	2	0.023	1.127	4.925	2.824	<0.001	1.776	4.491
TAPSE	0.580	0.653	0.054	6.223	-	-	-	-

B: Unstandardized regression coefficient, β: standardized regression coefficient, LAVI: left atrial volume index, LACI: left atrioventricular coupling index, CAD: coronary artery disease, LV EF: left ventricular ejection fraction, systolic excursion, TAPSE: tricuspid annular plane.

## Data Availability

The raw data supporting the conclusions of this article will be made available by the authors on request.
